# Evaluation of two frailty indices, with practical application in a vaccine clinical trial

**DOI:** 10.1080/21645515.2019.1622974

**Published:** 2019-06-21

**Authors:** Desmond Curran, Melissa K. Andrew, Myron J. Levin, Elisa Turriani, Sean Matthews, Charles Fogarty, Nicola P. Klein, Katrijn Grupping, Lidia Oostvogels, Kenneth E. Schmader

**Affiliations:** a GSK, Wavre, Belgium; b Division of Geriatric Medicine, Dalhousie University, Halifax, Nova Scotia, Canada; c Departments of Pediatrics and Medicine, University of Colorado, Anschutz Medical Campus, Aurora, CO, USA; d Freelance c/o GSK, Wavre, Belgium; e Spartanburg Medical Research, Spartanburg, SC, USA; f Kaiser Permanente Vaccine Study Center, Oakland, CA, USA; g Division of Geriatrics, Duke University Medical Center and GRECC, Durham Veterans Affairs Medical Center, Durham, NC, USA

**Keywords:** Varicella-zoster virus, herpes zoster, adjuvanted recombinant zoster vaccine, older adult, frailty, reactogenicity

## Abstract

Frail older adults are at increased risk of poor clinical outcomes. Frailty assessment is therefore important in clinical trials to understand the benefits and harms of interventions. However, consensus is lacking on how frailty should be assessed.

We developed a prospectively specified index using a battery of formal tests and instruments and a retrospectively generated index using medical comorbidities and patient reported outcomes (PROs) within an adjuvanted recombinant zoster vaccine (RZV) trial (NCT02979639). For both frailty indices (FIs), a total deficit score was calculated as the accumulation of deficits and participants were categorized as non-frail, pre-frail and frail. We assessed (1) the feasibility and validity of both FIs; (2) the impact of RZV vaccine reactogenicity by frailty status on Short Form-36 [SF-36] physical functioning (PF) scores.

Of 401 participants, aged ≥50 years, 236 (58.9%) were categorized non-frail, 143 (35.7%), pre-frail, and 22 (5.5%) frail using the prospective FI. Corresponding numbers for the retrospective FI were 192 (47.9%), 169 (42.1%) and 40 (10.0%), respectively. Strong concordance was observed between the frailty status assessments (*P* < .001). The proportion defined as frail increased from 1.5%, to 10.4% in participants aged 50–59, and ≥70 years, respectively, for the prospective FI. Corresponding numbers for the retrospective FI were 3.7%, and 17.2%, respectively. RZV vaccination was associated with a transient, non-clinically meaningful, decrease on the SF-36 PF score in frail participants.

Both frailty indices provided similar results. The retrospectively generated FI offers the advantage of being easier to incorporate into vaccine clinical trials of older adults.

## Introduction

Frailty is a term used in geriatric medicine to identify older adults who are at increased risk of poor clinical outcomes, such as disability, cognitive decline, falls, hospitalization, requiring long-term assisted care, or increased mortality.^1-3^ Frailty is defined as clinical vulnerability and is more predictive of health outcomes than chronological age. The aging process varies substantially between individuals because of unique features, such as genetic and environmental factors.^4^ For example, frailty may be influenced by an age-related decline in innate and adaptive humoral and cell-mediated immunity (i.e. immunosenescence), which impairs the ability to resist infection and respond to interventions such as vaccination.^5-8^ Regulatory guidelines have identified the importance of demonstrating that interventions available for vulnerable groups, such as frail individuals, should be fully and appropriately studied for their effects in these specific groups.^9^ This is of significance given the global trend of an aging population which will continue to increase global healthcare costs.^10^

Health care professionals, policy makers and researchers have not achieved consensus on how to define frailty and how to quantitate the grading of frailty.^3^ Mitnitski and Rockwood defined frailty status using a frailty index (FI) based on the accumulation of deficits (e.g. symptoms, signs, functional impairments, and laboratory abnormalities). This approach relies less on the nature of any deficit, but measures the cumulative effects of multiple deficits with age.^2^ Considering that this approach is relatively simple, the results yielded by FIs measured in this manner have been consistent between studies and surveys. This is the case even though not every FI considered the same deficits, or even the same number of deficits, as long as these meet certain criteria. These criteria include that the FI increases with age, correlates with adverse outcomes, is not saturating (i.e. developed a too high prevalence at younger ages), and represents a range of systems or domains.^1^ As such, the deficit accumulation approach offers the advantage of making frailty assessment accessible in the setting of clinical trials, if a robust frailty measure can be feasibly and reliably generated based on routinely collected data on health status and patient reported outcomes (PROs).

Although prior research on frailty focused on chronic conditions, general health status and physical frailty, more recently researchers have emphasized that frailty also involves psychological and social domains.^3^ In addition, some studies have incorporated PROs, such as the Short Form-36 (SF-36), into the definition of frailty. For example, Ryb et al defined frailty as having an SF-36 physical functioning (PF) score <75.^11^ Mansur defined frailty using weight loss and elements of the SF-36 scales as a measure of muscle weakness (SF-36 *PF score*), exhaustion (SF-36 *vitality score*), and physical inactivity (SF-36 *questions on frequency of physical activity*).^12^ Other studies have explored the correlation between the SF-36 and other FIs.^13,14^ In general, the SF-36 scales have correlated very strongly with other frailty measures.

Herpes zoster (HZ) is the clinical manifestation of the reactivation of latent varicella-zoster virus (VZV), which presents, usually in adults with impaired immunity, as a vesicular dermatomal rash frequently accompanied by pain.^15^ The incidence and severity of HZ increases with age, reflecting an age-related decline in VZV-specific T cell immunity.^16-18^ An adjuvanted recombinant HZ vaccine (RZV) containing glycoprotein E and the AS01_B_ adjuvant system is highly efficacious.^19,20^ Still, RZV is associated with local and systemic reactions. The ZOSTER-063 study (NCT02979639) was consequently designed as an open-label phase III trial to determine the impact of reactogenicity after administration of RZV on SF-36 and EuroQol-5 Dimension (EQ-5D) PRO measures in older adults.^21^ Frailty assessment following a previously described methodology was included in the study to evaluate possible inclusion in subsequent studies.^1,22,23^ As there is no consensus in the field on how to assess frailty we also undertook a post-hoc analysis to evaluate an alternative and more easily implementable FI, which used the PRO instruments assessed within the clinical trial paired together with the baseline medical history of vaccine recipients.

We report on two objectives: (1) to assess the feasibility and validity of two FIs: a prospectively specified index developed using a battery of formal tests and instruments; and a second retrospectively generated index incorporating medical comorbidities and PROs, (2) as a practical example, i.e. to determine the impact of reactogenicity on SF-36 PF and quality of life (QoL) after administration of the first dose of RZV as a function of the frailty status classification at baseline.

## Methods

### Study design and participants

Frailty assessment was incorporated into a phase III, single-arm, open-label study, conducted in 13 centers in the United States. Adults aged ≥50 years at enrolment received two 0.5 ml doses of RZV at months 0 and 2, administered intramuscularly. A summary of the exclusion criteria for the study is provided in the supplementary material. In this manuscript, we describe data up to 7 days post-dose 1. The study protocol was approved by the investigational review boards at each study center and was conducted in accordance with Good Clinical Practice and the Declaration of Helsinki. Further details regarding the study are provided elsewhere.^21^ Written informed consent was obtained from each participant prior to the performance of any study-specific procedures. The study is registered at http://www.clinicaltrials.gov (NCT02979639). The protocol is available at http://www.gsk-clinicalstudyregister.com (ID204928). Anonymized individual participant data and study documents are available for further research at www.clinicalstudydatarequest.com.

### Frailty assessments

#### Prospectively defined frailty index

A frailty assessment was performed 7 days before dose 1. The following domains were assessed:

- Disability: assessment of dependence on others to perform a list of specific daily activities (Supplementary Table S1, items 1 to 14);^1,22^
- Cognition: the Montreal Cognitive Assessment is a 30-point questionnaire measuring cognitive impairment (Supplementary Table S1, item 15);^24^
- Self-rating of health and change in health (Table S1, items 16 and 17);^1^
- Physical status: the SF-36 PF score was used as a measure of the physical activity of the study participant (Supplementary Table S1, item 18);^11^ and criteria for the definition of frailty developed by Fried et al.^25^ were utilized to categorize the physical frailty phenotype (Supplementary Table S1, items 19 to 21);
- Depression and exhaustion: the Center for Epidemiologic Studies Depression Scale – Revised was used to screen for depression and depressive disorders (Supplementary Table S1, item 22);^26^
- Multimorbidity: occurrence of 14 medical history conditions was based on the work of Song et al.^23^ (Supplementary Table S1, items 23 to 36);

Further details on the deficits assessed and the scoring of the FI components are provided in Supplementary Table S1. A total deficit score was calculated as the accumulation of the individual deficits ranging from 0 to 36. The FI was then calculated by converting the deficit score into an index from 0 to 1 as follows:

- FI = (deficit score/n)

where n is the number of non-missing components of the 36 items. Each study participant was then assigned to one of three categories based on the FI as follows: FI≤0.08 is classified as non-frail; 0.08< FI≤0.25 is classified as pre-frail; FI>0.25 is classified as frail.^23^

#### Retrospectively generated frailty index

A retrospectively generated frailty assessment was based on a combination of the occurrence of specific age dependent diseases and data from two PRO questionnaires (SF-36 and EQ-5D) (see Table 1).^23,27,28^ The SF-36 is a multi-purpose health survey comprising 36 questions, including scales for PF, Role Physical, Bodily Pain, General Health, Vitality, Social Function, Role Emotional, and Mental Health.^27^ EQ-5D is a generic measure of health status that defines health in terms of mobility, self-care, usual activities, pain/discomfort, and anxiety/depression. These five items are combined to generate health profiles, which are converted to a single index utility score, where a higher score represents a better QoL.^28^ Both questionnaires were completed by study participants at Visit 1 (7 days pre-dose 1), Visit 2 (immediately before dose 1) and Visit 3 (7 days post-dose 1). Additionally, all participants completed the SF-36 PF component (questions 3a-3j) and the entire EQ-5D questionnaire at home daily for 6 days post-dose 1.

**Table 1.** Detail of SF-36 and EQ-5D components contribution to the retrospective frailty index.

Item	Scoring method based on response to question	Maximum Contribution to Frailty Index
*SF-36 Q1* (General Health)	Poor = 1 Fair = 0.5 Good = 0 Very good = 0 Excellent = 0	1
*SF-36* Q11A-11D (General Health)	**Q11A, Q11C** Definitely true = 1 Mostly true = 0.5 Don’t know = 0 Mostly false = 0 Definitely false = 0 **Q11B, Q11D** Definitely true = 0 Mostly true = 0 Don’t know = 0 Mostly false = 0.5 Definitely false = 1	4
*SF-36* Q3I – Q3J (Physical functioning)	Limited a lot = 1 Limited a little = 0.5 Not limited at all = 0	10
*SF-36* Q9A – Q9I (Vitality and Mental health)	**Q9A, Q9D, Q9E, Q9H** All of the Time = 0 Most of the time = 0 Some of the time = 0 A little of the time = 0.5 None of the time = 1 **Q9B, Q9C, Q9F, Q9G, Q9I** All of the Time = 1 Most of the time = 0.5 Some of the time = 0 A little of the time = 0 None of the time = 0	9
*SF-36* Q2 Compared to one week before, how did the subject rate his/her health in general?	Much worse = 1 Somewhat worse = 0.5 Same = 0 Somewhat better = 0 Better = 0	1
*EQ-5D* Mobility	No Problems = 0 Some Problems = 0.5 Confined to bed = 1	1
*EQ-5D* Anxiety	No Anxiety = 0 Moderate Anxiety = 0.5 Extreme Anxiety = 1	1
*EQ-5D* Self care	No Problems = 0 Some Problems = 0.5 Inability to wash or dress himself/herself = 1	1
*EQ-5D* Usual activities	No Problems = 0 Some Problems = 0.5 Inability to perform usual activities = 1	1
**Total**		**29**

As in the prospectively defined frailty assessment described above, 14 comorbidities were assessed (see supplementary Table S1, items 23 to 36). In addition to the study participant’s medical comorbidity which contributes a maximum score of 14, the pre-vaccination SF-36 and EQ-5D questionnaires contribute a maximum score of 25 and 4, respectively (see Table 1). Consequently, a total deficit score is calculated as the accumulation of the individual’s deficits, ranging from 0 to 43. As per the prospectively specified FI, the retrospectively generated FI was calculated by converting the deficit score into an index from 0 to 1 (i.e. FI = (deficit score/n) where n is the number of non-missing components of the 43 items) and frailty status was defined using the same cut-off points (i.e. 0.08 and 0.25).

Distributions and properties of both FIs were explored using descriptive methods. The association of both FIs with (1) age as both a categorical variable (i.e. age groups 50–59, 60–69 and ≥70 years) and as a continuous variable (assuming both a linear and an exponential distribution), and (2) the SF-36 PF score and the EQ-5D utility score were explored using descriptive statistics. The Kendall’s tau-b test was used to assess the concordance between the frailty status assessed using the 2 different methods. We investigated if any variable included in the FI saturated.

The secondary objective of this manuscript was to assess the impact of RZV vaccine reactogenicity on QoL, which was a pre-specified endpoint of the study protocol. We presented the mean SF-36 PF score and mean EQ-5D utility score by days post-dose 1 for the prospectively specified frailty status. A change of 3.3 in SF-36 PF score and a change of 0.074 in the EQ-5D utility score are considered clinically relevant.^29,30^

## Results

### Participants

401 participants received the first dose of RZV. Participants had a mean age of 64.6 years, and, as pre-defined, were equally distributed between three age groups: 50–59 years (33.4%), 60–69 years (33.2%), ≥70 years (33.4%). Most participants were Caucasian (82.8%) and there were more females than males (58.6 vs. 41.4%). Further details regarding the study results post-dose 1 are provided elsewhere.^21^

### Prospectively defined frailty index

Overall, 236 (58.9%) participants were categorized as non-frail, 143 (35.7%) as pre-frail and 22 (5.5%) as frail. Table 2 presents the frailty status by age; 1.5% of participants 50–59 years of age (YOA) were considered frail, compared with 4.5% of those 60–69 YOA, and 10.4% of those ≥70 YOA.

**Table 2.** Distribution of age, SF-36 physical functioning score and EQ-5D scores by frailty status according to frailty index.

		Non-frail N = 236	Pre-frail N = 143	Frail N = 22
Prospectively generated FI		n (%)	n (%)	n (%)
Age	50–59 YOA	111 (82.8)	21 (15.7)	2 (1.5)
	60–69 YOA	80 (60.2)	47 (35.3)	6 (4.5)
	≥70 YOA	45 (33.6)	75 (56.0)	14 (10.4)
		Statistic	Statistic	Statistic
SF-36	Mean	90.7	71.6	40.7
physical	Standard deviation	13.18	23.59	19.84
functioning	Median	95.0	75.0	35.0
	Interquartile range	(90.0–100.0)	(55.0–90.0)	(30.0–50.0)
	Range	(25.0–100.0)	(5.0–100.0)	(5.0–95.0)
EQ-5D	Mean	0.922	0.841	0.672
	Standard deviation	0.0960	0.1408	0.1633
	Median	1.000	0.827	0.699
	Interquartile range	(0.827–1.000)	(0.800–1.000)	(0.597–0.778)
	Range	(0.467–1.000)	(0.378–1.000)	(0.308–1.000)
		Non-frail N = 192	Pre-frail N = 169	Frail N = 40
Retrospectively generated FI		n (%)	n (%)	n (%)
Age	50–59 YOA	95 (70.9)	34 (25.4)	5 (3.7)
	60–69 YOA	64 (48.1)	57 (42.9)	12 (9.0)
	≥70 YOA	33 (24.6)	78 (58.2)	23 (17.2)
		Mean (SD)	Mean (SD)	Mean (SD)
SF-36 physical functioning	Physical Function	96.4 (4.49)	75.3 (16.47)	42.8 (18.44)
	Role Physical	97.6 (6.03)	80.5 (18.21)	53.7 (25.55)
	Bodily Pain	86.6 (12.23)	70.9 (18.76)	51.5 (25.57)
	General Health	86.9 (9.44)	73.1 (13.89)	52.7 (20.45)
	Vitality	80.6 (11.24)	68.5 (13.65)	44.8 (19.91)
	Social Functioning	97.5 (7.21)	91.4 (14.03)	71.1 (26.88)
	Role Emotional	98.1 (5.65)	90.6 (13.88)	66.5 (25.58)
	Mental Health	89.1 (8.10)	83.2 (13.00)	70.1 (19.50)
EQ-5D	Utility Score	0.957 (0.0664)	0.869 (0.0916)	0.699 (0.1690)
Deficits	Comorbidities	0.94 (0.84)	2.52 (1.35)	4.00 (1.84)
	SF-36	0.47 (0.55)	3.10 (1.73)	8.94 (2.42)
	EQ-5D	0.06 (0.20)	0.37 (0.43)	1.24 (0.70)
	Total	1.47 (0.99)	5.99 (2.06)	14.2 (2.73)

N = total number of participants by frailty category; n (%) = number (percentage) of participants by age group; YOA = years of age; SD = standard deviation; SF-36 = Short Form-36; EQ-5D = EuroQol-5 Dimension

The mean SF-36 PF scores at Day −7 were 90.7 (95% confidence interval [CI]: 89.0–92.4) in non-frail participants, 71.6 (95% CI: 67.7–75.5) in pre-frail participants and 40.7 (95% CI: 32.4–49.0) in frail participants. The mean EQ-5D utility scores at Day −7 were 0.92 (95% CI: 0.91–0.93) in non-frail participants, 0.84 (95% CI: 0.82–0.86) in pre-frail participants and 0.67 (95% CI: 0.60–0.74) in frail participants.

### Retrospectively generated frailty index

**Figure 1.**
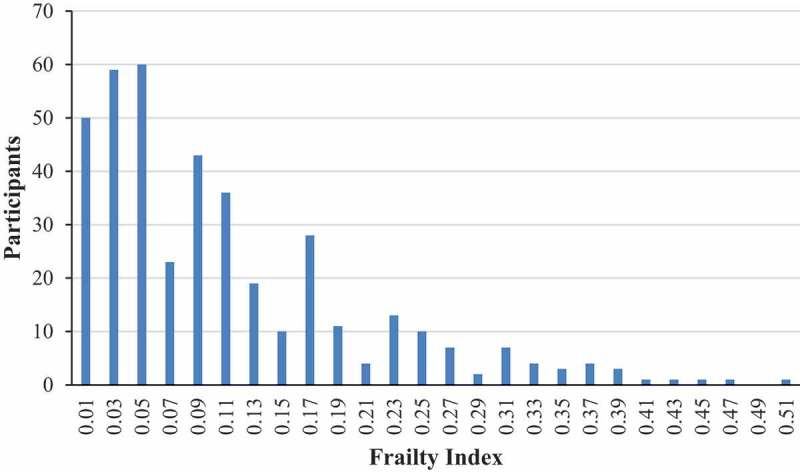
Distribution of the retrospectively generated frailty index.

Figure 1 presents the distribution of the retrospectively generated FI score, which follows a gamma distribution tailing to the right with a maximum score of 0.50. As such, there is a consistent, submaximal upper limit to the percentage of deficits that any person can accumulate. Thirty-four individuals had a score of 0, while the median and mean were 0.08 and 0.11, respectively. Overall, 192 (47.9%) participants were categorized as non-frail, 169 (42.1%) as pre-frail and 40 (10.0%) as frail. Table 2 presents the frailty status by age; 3.7% of participants 50–59 YOA were considered frail, compared with 9.0% of those 60–69 YOA, and 17.2% of those ≥70 YOA.

The mean SF-36 PF scores at Day −7 were 96.4 (95% CI: 95.8–97.0) in non-frail participants, 75.3 (95% CI: 72.8–77.8) in pre-frail participants and 42.8 (95% CI: 37.0–48.6) in frail participants. The mean EQ-5D utility scores were 0.96 (95% CI: 0.95–0.97) in non-frail participants, 0.87 (95% CI: 0.86–0.88) in pre-frail participants and 0.70 (95% CI: 0.66–0.74) in frail participants.

### Comparison of frailty indices

A higher number of subjects were defined as frail using the retrospectively defined frailty index compared to the prospectively defined index. For both indices, the majority of subjects who were considered frail were aged ≥ 70 years, i.e. 63.6% and 57.5% for the prospectively and retrospectively defined frailty indices, respectively (see Table 2). Although some differences can be seen between the two indices presented in Table 2, a similar trend was observed for both indices demonstrating that the SF-36 and EQ-5D scores decreased consistently with increasing frailty, and the number of deficits and the presence of a comorbidity deficit (see Supplementary Tables S2 and S3) increased consistently with increasing frailty. Both Supplementary Tables S2 and S3 demonstrate that high blood pressure was the most prevalent comorbidity with 202 (50.4%) participants reporting the condition. This suggests that no variable included in the FI was saturated.

Table 3 compares the prospectively specified and retrospectively defined frailty categorizations. Note, both frailty status assessments were available for all 401 subjects who received the first dose of RZV. In total, 304 (75.8%) participants were assigned to the same retrospective and prospective frailty categories. Of the 236 study participants classified as non-frail with the prospective FI, only 1 was classified as frail using the retrospective FI. The Kendall’s tau-b statistic was significant (*P* < .001), indicating strong concordance between the frailty status assessed using the two different methods.

**Table 3.** Comparison of frailty status according to the retrospectively generated vs. prospectively specified frailty status.

Prospective	Non-frail	Pre-frail	Frail	Total prospective frailty
Retrospective	n (%)	n (%)	n (%)	n (%)
Non-frail	178 (75.4)	57 (24.2)	1 (0.4)	236 (58.9)
Pre-frail	14 (9.8)	108 (75.5)	21 (14.7)	143 (35.7)
Frail	0 (0.0)	4 (18.2)	18 (81.8)	22 (5.5)
Total retrospective frailty	192 (47.9)	169 (42.1)	40 (10.0)	401 (100)

n (%) = number (percentage) of participants by frailty status

**Figure 2.**
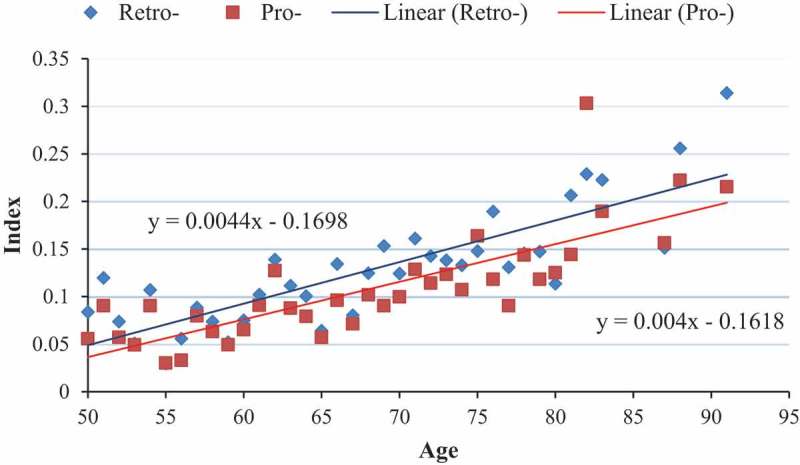
Mean frailty index by age and type of frailty index, assuming a linear model.

Figure 2 presents a scatterplot of the mean FI by age and type of FI. The intercept was −0.1618 and −0.1698 for the prospective and retrospective frailty indices, respectively. The indices increased on average by 0.0040 and 0.0044, respectively, each year. By the age of 90, the mean FI was approximately 0.2 for both indices, suggesting that most participants who are older are either frail or pre-frail. The linear equations presented in Figure 2 demonstrated a good fit for both the prospective and retrospective frailty indices (i.e. R-squared ≥0.60, *P* < .001). Figure S1 presents the same results assuming an exponential distribution resulting in a similar fit for both the prospective and retrospective frailty indices (i.e. R-squared ≥0.60, *P* < .001).

Pro-: Prospectively specified, Retro-: Retrospectively generated

**Figure 3.**
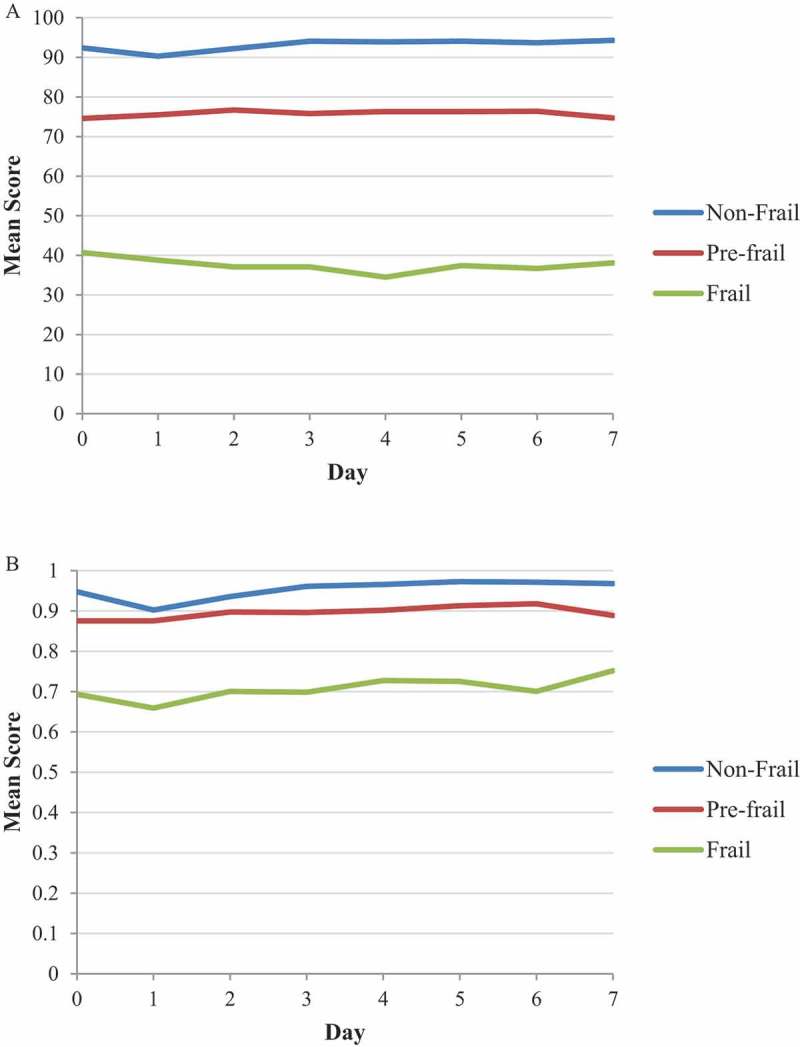
Mean SF-36 Physical functioning score (Panel A) and Mean EQ-5D Utility score (Panel B) by days post-dose 1 by prospectively specified frailty status.

As per the secondary objective of this manuscript, Figure 3 presents the mean SF-36 PF score and mean EQ-5D utility score by day post-dose 1 by frailty status for the prospectively specified FI. There was a small (i.e. non-clinically meaningful) transient decrease in the SF-36 PF score in non-frail participants, i.e. the score decreased from 92.4 on Day 0 to 90.3 on Day 1, but had returned to baseline by Day 2 (i.e. 92.2). No apparent decrease was observed in SF-36 PF score in pre-frail participants, whereas, there was a gradual decrease until Day 4 with a subsequent increase until Day 7 for frail participants. The mean EQ-5D utility score decreased by 0.045 in non-frail participants and 0.035 in frail participants on Day 1, with values returning to baseline by Day 2. These decreases were considered not clinically relevant.^30^ No apparent decrease was observed in EQ-5D utility score in pre-frail participants.

## Discussion

We present the results from both a prospectively specified and a retrospectively generated FI. Both frailty assessments were completed for all 401 participants who received the first dose of RZV in the study. The frailty status generated from both FI differentiated participants by age, SF-36 PF score and EQ-5D utility scores. The frailty status based on the retrospective analysis of the comorbidity status and SF-36 and EQ-5D scores demonstrated strong concordance with the prospective frailty status that was based on a previously validated methodology. We demonstrated that none of the comorbidity status items saturated. For the SF-36 and EQ-5D items, although ceiling effects may be present, it is unlikely that these measures saturate for the general population at younger ages.

As a secondary objective of this manuscript we demonstrated using a practical application, how subjects can be classified into frail categories to investigate clinical outcomes. The study results suggested that reactogenicity associated with RZV had a transient, non-clinically meaningful, impact on SF-36 PF and EQ-5D utility scores in frail participants. Frailty assessment will become more important in the future to characterize populations for which new therapies or vaccines are being developed, since many such products will target older adults and frail populations. It is therefore important to note that even without specific attempts to recruit frail subjects, we identified pre-frail and frail participants, which demonstrates that if relatively nonrestrictive in/exclusion criteria are applied (see supplementary material for details on the exclusion criteria), one can expect a relatively broad range of participants.

A complete evaluation (i.e. a ‘gold standard’) of frailty requires a multidimensional, interdisciplinary assessment, including domains such as physical and cognitive function, nutritional status, multimorbidity, concomitant medications, and socio-economic factors.^9^ The recent European Medicines Agency (EMA) guidelines focus primarily on physical frailty, recognizing that due to the complexity of frailty assessment, a complete evaluation was beyond the scope of the guidelines. In practice, including a complete evaluation of frailty assessment in a clinical study is complex and burdensome for study participants and investigators, and may impact the feasibility of the study. This is particularly true for phase III vaccine clinical trials where thousands of participants are recruited and followed to observe a predefined number of cases who develop disease. In this study we explored the feasibility of including a prospective FI, to establish a workable (from an operational perspective) methodology.

We proposed additionally a FI based on medical comorbidities that are generally collected as part of the medical history in clinical studies, in association with standard PRO instruments (i.e., SF-36 and EQ-5D). Our work shows that it can be used retrospectively in studies already including the SF-36 and EQ-5D questionnaires. Although the proposed retrospective index may have limitations, it can also be implemented prospectively in clinical trials conducted in older adults.

The EMA guidelines emphasize that treatments for vulnerable groups, such as frail individuals, should be appropriately studied for unique treatment effects. Therefore, it is important to be able to characterize the frailty level of the study population, either descriptively, or to allow a targeted or stratified recruitment of study participants with different levels of frailty. Our work confirms this is feasible, via 2 different methods of FI. Clinical events such as a fall or the onset of diseases such as influenza may result in a decline of physical function in frail individuals.^31^ As such, it is also important to study the impact of preventive interventions (e.g. diet, exercise, fall prevention and vaccination) on the reduction of risk of individuals becoming frailer, thereby promoting active and healthy aging.^32,33^ The growth of this vulnerable population increases the need for closer attention to long-term care policies and public health decision-making.

Our study is not without limitations. We assessed feasibility based on our ability to define frailty status for all subjects. We did not ask the investigators or subjects about how burdensome the work was, etc. Our frailty measures are based on self-reported variables rather than being based on objective measurements. However, frailty indices based on self-reporting have been shown to be valid across many settings, and the approach represents a means of generating a holistic measure of health and vulnerability that explicitly considers the participant’s experience (i.e. of symptoms, functional impacts) in clinical trials.^1,2,7,22,23^

Traditionally, research on frailty focused on chronic conditions, general health status and physical frailty. Commonly used methods of assessing physical frailty are the Short Physical Performance Battery (SPPB),^34^ which measures three separate tests (i.e. standing balance, gait speed, and ability to rise from a chair) and the Fried Frailty phenotype which assesses five criteria exploring the presence/absence of physical signs or symptoms (i.e. involuntary weight loss, exhaustion, slow gait speed, poor handgrip strength, and sedentary behavior).^25^ However, some researchers have suggested that the required contact between the individual and the assessor for formally assessing physical frailty may suggest that other assessments (e.g. self-reported questionnaires) may be preferred in the first estimation of the individual’s frailty profile.^35^ More recently, researchers have emphasized that frailty also involves psychological and social domains.^3^ In addition to the medical comorbidities and SF-36 PF, the proposed index included items from both the SF-36 and EQ-5D questionnaires assessing general health (e.g. ‘my health is excellent’, ‘I get sick easier than other people’, ‘I expect my health to get worse’), vitality (e.g. feeling full of life, calm, peaceful) and mental health (e.g. feeling depressed, anxious and worn out).

In summary, we used information gathered in our study to assess the feasibility and validity of two FIs. Both indices provided similar results, although from a feasibility perspective, the FI generated retrospectively using medical comorbidities and PROs, can be more easily incorporated as an assessment in future clinical trials conducted in older adults. In addition, we demonstrated that reactogenicity associated with dose 1 of RZV had a transient, non-clinically meaningful, impact on SF-36 PF and EQ-5D utility scores in frail participants.

## Abbreviations

EQ-5D: EuroQol-5 Dimension
FI: frailty index
HZ: herpes zoster
PF: physical functioning
PROs: patient reported outcomes
QoL: quality of life
RZV: adjuvanted recombinant herpes zoster vaccine
SF-36: Short Form-36
VZV: varicella-zoster virus
