# Supplementary material for: Evaluation of two frailty indices, with practical application in a vaccine clinical trial
Source: Hum Vaccin Immunother. 2019 Jun 21;15(12):2960–8. doi: 10.1080/21645515.2019.1622974 (PMC6930102; doi:10.1080/21645515.2019.1622974)
Supplement: Supplemental Material [file khvi-15-12-1622974-s001.docx]

Supplementary Material

Evaluation of two frailty indices,

with practical application in a

Vaccine clinical trial

Desmond Curran, Melissa K. Andrew, Myron J. Levin,

Elisa Turriani, Sean Matthews, Charles Fogarty, Nicola P. Klein, Katrijn Grupping, Lidia Oostvogels & Kenneth E. Schmader

Human Vaccines & Immunotherapeutics

**Supplementary materials**

**Exclusion criteria**

Participants were excluded if they had a history of herpes zoster (HZ), had received or planned to receive another HZ vaccine during the study; had received immunoglobulin or blood products within the previous three months or were scheduled to receive these products during the study period; had received immunosuppressants or other immune-modifying drugs within the previous six months (including chronic corticosteroids, long-acting immune-modifying agents or immunosuppressive/cytotoxic therapy); had received/planned to receive any live vaccine within 30 days of the adjuvanted HZ subunit vaccine (HZ/su). Women who were pregnant, lactating or planning to become pregnant were ineligible to participate.

**Table S1.** Details of components of prospectively specified frailty index

| **Item No** | **Category** | **Response** | **Mapped Score as used in Frailty Index** |
| --- | --- | --- | --- |
| 1 | Help Bathing | Yes, No | Yes=1, No=0 |
| 2 | Help Dressing | Yes, No | Yes=1, No=0 |
| 3 | Help getting in or out of chair | Yes, No | Yes=1, No=0 |
| 4 | Help walking in or around house | Yes, No | Yes=1, No=0 |
| 5 | Help eating | Yes, No | Yes=1, No=0 |
| 6 | Help Grooming | Yes, No | Yes=1, No=0 |
| 7 | Help using Toilet | Yes, No | Yes=1, No=0 |
| 8 | Help up/down stairs | Yes, No | Yes=1, No=0 |
| 9 | Help lifting | Yes, No | Yes=1, No=0 |
| 10 | Help shopping | Yes, No | Yes=1, No=0 |
| 11 | Help with housework | Yes, No | Yes=1, No=0 |
| 12 | Help with meal preparation | Yes, No | Yes=1, No=0 |
| 13 | Help taking medications | Yes, No | Yes=1, No=0 |
| 14 | Help with finances | Yes, No | Yes=1, No=0 |
| 15 | Cognitive score (MoCA) | Range 0 to 30 | Mapped to a score between 0 and 1 according to the following criteria:  If response <6 then mapped score=1 (Severe Dementia)  If 6≤ response≤11 then mapped score=0.75 (Moderate Dementia)  If 12≤response≤18 then mapped score=0.5 (Mild Dementia)  If 19≤response≤25 then mapped score=0.25 (Mild Cognitive impairment)  If response≥26 then mapped score=0 (No Cognitive impairment) |
| 16 | Self-Rating of Health | Poor, Fair, Good, Very Good, Excellent | If rating=Poor then mapped score=1  If rating=Fair then mapped score=0.75  If rating=Good then mapped score=0.5  If rating=Very Good then mapped score=0.25  If rating=Excellent then mapped score=0 |
| 17 | Health Change in last year | Worse, Better/Same | If change=Worse then mapped score=1  Else mapped score=0 |
| 18 | SF-36 Physical Functioning Score | Derived score for items 3A to 3J. (Range: 0 - 100) | If derived score <50 then mapped score=1  If derived score ≥50 and <75 then mapped score=0.5  If derived score ≥75 then mapped score=0 |
| 19 | Unintentional weight loss | Yes, No | Yes=1, No=0 |
| 20 | Weakness (grip strength) | Yes, No | Yes=1, No=0 |
| 21 | Slow Walking Speed | Yes, No | Yes=1, No=0 |
| 22 | Depression score (CESD-R) | Range 0 to 60 | Mapped to a score between 0 and 1 according to the following criteria:  If score <16 then mapped score=0  If 16≤score≤21 then mapped score=0.5  If score >21 then mapped score=1 |
| 23 | High Blood pressure, | Yes, No | Yes=1, No=0 |
| 24 | Heart Attack, | Yes, No | Yes=1, No=0 |
| 25 | Heart Failure, | Yes, No | Yes=1, No=0 |
| 26 | Cerebrovascular Disease, | Yes, No | Yes=1, No=0 |
| 27 | Cancer, | Yes, No | Yes=1, No=0 |
| 28 | Diabetes Mellitus | Yes, No | Yes=1, No=0 |
| 29 | Arthritis, | Yes, No | Yes=1, No=0 |
| 30 | Chronic Lung Disease, | Yes, No | Yes=1, No=0 |
| 31 | Long Term disability or Handicap | Yes, No | Yes=1, No=0 |
| 32 | Stomach or Intestinal Ulcers | Yes, No | Yes=1, No=0 |
| 33 | Migraine | Yes, No | Yes=1, No=0 |
| 34 | Cataract | Yes, No | Yes=1, No=0 |
| 35 | Hearing Problems | Yes, No | Yes=1, No=0 |
| 36 | Glaucoma | Yes, No | Yes=1, No=0 |

**Table S2.** Distribution of comorbidities by the prospectively generated frailty status

|  |  | Non-Frail  N = 236 | Pre-Frail  N = 143 | Frail  N = 22 |
| --- | --- | --- | --- | --- |
|  | | n (%) | n (%) | n (%) |
| High Blood Pressure | | 71 (30.1) | 112 (78.3) | 19 (86.4) |
| Heart Attack | | 1 (0.4) | 17 (11.9) | 5 (22.7) |
| Heart Failure | | 0 (0.0) | 9 (6.3) | 4 (18.2) |
| Cerebrovascular Disease | | 4 (1.7) | 15 (10.5) | 6 (27.3) |
| Cancer | | 19 (8.1) | 30 (21.0) | 8 (36.4) |
| Diabetes Mellitus | | 11 (4.7) | 59 (41.3) | 11 (50.0) |
| Arthritis | | 57 (24.2) | 81 (56.6) | 17 (77.3) |
| Chronic Lung Disease | | 10 (4.2) | 19 (13.3) | 6 (27.3) |
| Long term Disability or Handicap | | 2 (0.8) | 6 (4.2) | 5 (22.7) |
| Stomach or Intestinal Ulcers | | 7 (3.0) | 5 (3.5) | 2 (9.1) |
| Migraine | | 18 (7.6) | 12 (8.4) | 3 (13.6) |
| Cataract | | 15 (6.4) | 35 (24.5) | 8 (36.4) |
| Hearing problems | | 17 (7.2) | 22 (15.4) | 7 (31.8) |
| Glaucoma | | 1 (0.4) | 9 (6.3) | 2 (9.1) |
| None | | 76 (32.2) | 2 (1.4) | 0 (0.0) |

N = total number of participants by frailty category; n (%) = number (percentage) of participants by comorbidity

**Table S3.** Distribution of comorbidities by the retrospectively generated frailty status

|  |  | Non-Frail  N = 192 | Pre-Frail  N = 169 | Frail  N = 40 |
| --- | --- | --- | --- | --- |
|  | | n (%) | n (%) | n (%) |
| High Blood Pressure | | 58 (30.2) | 112 (66.3) | 32 (80.0) |
| Heart Attack | | 1 (0.5) | 13 (7.7) | 9 (22.5) |
| Heart Failure | | 0 | 5 (3.0) | 8 (20.0) |
| Cerebrovascular Disease | | 3 (1.6) | 12 (7.1) | 10 (25.0) |
| Cancer | | 16 (8.3) | 31 (18.3) | 10 (25.0) |
| Diabetes Mellitus | | 10 (5.2) | 52 (30.8) | 19 (47.5) |
| Arthritis | | 39 (20.3) | 90 (53.3) | 26 (65.0) |
| Chronic Lung Disease | | 9 (4.7) | 17 (10.1) | 9 (22.5) |
| Long term Disability or Handicap | | 1 (0.5) | 5 (3.0) | 7 (17.5) |
| Stomach or Intestinal Ulcers | | 3 (1.6) | 9 (5.3) | 2 (5.0) |
| Migraine | | 17 (8.9) | 11 (6.5) | 5 (12.5) |
| Cataract | | 11 (5.7) | 34 (20.1) | 13 (32.5) |
| Hearing problems | | 12 (6.3) | 27 (16.0) | 7 (17.5) |
| Glaucoma | | 1 (0.5) | 8 (4.7) | 3 (7.5) |
| None | | 68 (35.4) | 9 (5.3) | 1 (2.5) |

N = total number of participants by frailty category; n (%) = number (percentage) of participants by comorbidity

**Figure S1.** Mean frailty index by age and type of frailty index, assuming an exponential model.

Pro-: Prospectively specified, Retro-: Retrospectively generated
